# Functional Characterization of the M36 Metalloprotease FgFly1 in *Fusarium graminearum*

**DOI:** 10.3390/jof8070726

**Published:** 2022-07-12

**Authors:** Xintong Wang, Miaomiao He, Huanhuan Liu, Huiyi Ding, Kouhan Liu, Ying Li, Peng Cheng, Qiang Li, Baotong Wang

**Affiliations:** State Key Laboratory of Crop Stress Biology for Arid Areas, College of Plant Protection, Northwest A & F University, Yangling 712100, China; xintongwang@nwafu.edu.cn (X.W.); hemiaomiao@nwsuaf.edu.cn (M.H.); lhhlxx0323@nwafu.edu.cn (H.L.); dinghy@nwafu.edu.cn (H.D.); liukouhan6@nwafu.edu.cn (K.L.); liying2018@nwafu.edu.cn (Y.L.); pengcheng@nwafu.edu.cn (P.C.)

**Keywords:** *Fusarium graminearum*, FgFly1, wheat, CAMTA

## Abstract

Fungalysin metallopeptidase (M36), a hydrolase, catalyzes the hydrolysis of alanine, glycine, etc. Normally, it is considered to play an important role in the progress of fungal infection. However, the function of fungalysin metallopeptidase (M36) in *Fusarium graminearum* has not been reported. In this study, we explored the biological functions of FgFly1, a fungalysin metallopeptidase (M36) of *F. graminearum*. We found that *ΔFgFly1* did not affect the ability to produce DON toxin, although it inhibited spore germination during asexual reproduction and reduction in pathogenicity compared with PH-1. Therefore, we speculated that FgFly1 affects the pathogenicity of *F.graminearum* by affecting pathways related to wheat disease resistance. Target protein TaCAMTA (calmodulin-binding transcription activator) was selected by a yeast two-hybrid (Y2H) system. Then, the interaction between FgFly1 and TaCAMTA was verified by bimolecular fluorescent complimentary (BiFC) and luciferase complementation assay (LCA). Furthermore, compared with wild-type *Arabidopsis thaliana*, the morbidity level of *ΔAtCAMTA* was increased after infection with *F.* *graminearum*, and the expression level of *NPR1* was significantly reduced. Based on the above results, we concluded that FgFly1 regulated *F. graminearum* pathogenicity by interacting with host cell CAMTA protein.

## 1. Introduction

Wheat, an important food crop worldwide, is threatened by various diseases during its life cycle. Fusarium head blight (FHB) is a worldwide disease caused by Fusarium fungi, which can result in a yield loss of more than 30% in epidemic years [1,2,3]. Moreover. the development of FHB is accompanied by the accumulation of two main mycotoxins, deoxynivalenol (DON) and zearalenone (ZEA) [4,5], which can significantly reduce the quality of grains, threatening the health of humans and livestock [6]. At present, management of FHB remains challenging, mainly because a germplasm with complete resistance or immunity to *F. graminearum* is lacking, and the main control method for *F. graminearum* is currently the use of azole fungicides during the flowering stage of wheat. However, these methods are costly and time-consuming. Therefore, it is essential to explore the pathogenic and molecular mechanisms of *F. graminearum* [7]. 

When a pathogen invades a host plant, the pathogen secretes effectors into the plant, which induces a series of disease-resistance responses [8]. In a narrow sense, the effectors in phytopathogenic fungi are the proteins secreted from pathogens into extracellular and intracellular spaces of host plants [9,10,11,12]. A generalized definition of effectors is “proteins and small molecules that alter host cell structures and functions, thereby facilitating the colonization of pathogens” [13].

A common strategy of pathogenic fungi is to degrade host structural barriers by secreting extracellular enzymes [14,15,16]. Elastase metalloproteinase plays an important role in the progress of fungal invasion [17]. Metalloelastase is a kind of protease that catalyzes the hydrolysis of carboxyl-containing polypeptide bonds. Its active center is dependent on metal ions [18]. Metalloproteinase and elastinolytic serine proteinase have been found in *Aspergillus fumigatus* and *Aspergillus flavus*, and they are important pathogenic factors of *Aspergillosis* [16,17,18,19,20,21,22,23]. It is evident that elastase metalloproteinase is a key virulence factor; following the loss of the serine protease gene, *Aspergillus fumigatus* are still virulent, which may be compensated by other proteases [16]. 

Metalloproteases are proteases that contain metal ions in their active centers. Zinc metalloproteases, i.e., Zn^2+^-containing proteases, are widely distributed in eukaryotes and prokaryotes and are divided into four major groups, among which those containing zinc ion-binding motifs are called the zincin superfamily. This superfamily is further divided into more than ten families depending on the third zinc ion-binding site [24]. The biggest difference between metalloproteases and threonine proteases, serine proteases, aspartate proteases and cysteine proteases is that the active region contains one or more metal ions; in addition, thermolysin contains four Ca^2+^ ions, two of which are located near the active center, with the two others located on the surface of the two loops; these Ca^2+^ ions are vital for stabilizing the protease structure and preventing enzyme autolysis [25]. According to the binding sites of Zn^2+^, metalloproteases can be classified into snake venom metalloproteases, thermolysin, Serratia marcescens proteases, lobster peptidases and matrix metalloproteases (MMPs) [24]. Metalloproteases secreted by pathogens are associated with the virulence of the fungus itself [26]. For example, in the early stages of Bacillus anthracis infection, the secreted fungal toxin metalloprotease gene cgf1 is significantly upregulated, suggesting an important regulatory role in fungus–plant interactions [27,28]. In contrast, in the fungal disease *Pyricularia oryzae*, the zinc metalloprotease Avr-Pita triggers a signal transduction pathway recognized by the cytoplasmic receptor Pita [29].

Calmodulin-binding transcription activators (CAMTAs) are a class of proteins that bind calmodulin and activate transcription. As early as 2000, Yang and Poovaiah isolated a CaMBP from *Nicotiana tabacum* seedlings, called ethylen response 1 (NtER1) because of its rapid expression induced by ethylene, which is a CAMTA protein [30]. In 2002, Bouché discovered a CAMTA protein in rapeseed and revealed that there are 1, 2 and 6 CAMTA proteins in multicellular organisms such as *Drosophila melanogaster*, *Homo sapiens* and *A.thaliana*, respectively [31]. The CAMTA protein OsCBT (*Oryza sativa* CaM-binding transcription factor) was identified in rice, and six other homologous proteins of OsCBT were also found [32]. In addition, seven SlSR/CAMTA genes were successfully cloned during fruit development and ripening in *Solanum lycopersicum* [33].

As a transcriptional activator, the transcriptional activation region of *A. thaliana* AtCAMTA1 is located between GC-1 and TIG, and calmodulin-binding activation is dependent on Ca^2+^ [31,34]. Both T-DNA insertion mutants of *AtCAMTA3*, *camta3-1* and *camta3-2* exhibit the phenotype of slow growth, dwarf plants and yellow rosette leaves. Microarray analysis show that 6 genes were downregulated and 99 genes were upregulated. It is noteworthy that 32 of the upregulated genes are related to plant disease resistance, such as *WRKY33*, *PR1* and chitinase genes. Infection experiments showed that both *camta3-1* and *camta3-2* were more resistant to bacteria (*Pseudomona syringae* pv. tomato DC3000) and fungi (*Botrytiscinerea*) than wild type. Consistent with this, the content of H_2_O_2_ in the mutant plants was higher than that in wild-type plants. These results showed that AtCAMTA3 was involved in the regulation of plants’ defense response [35]. Du et al. (2009) found that mutations in *AtSR1/CAMTA3* resulted in an increase in salicylic acid (SA) content and subsequently enhanced disease resistance in *A. thaliana* [36]. As a CaM-binding protein, CAMTA (calmodulin-binding transcription activator) responds to a series of environmental stresses, such as drought, salt and cold, as well as hormonal signals, such as abscisic acid, ethylene and auxin. CAMTA plays an important regulatory role in plant biotic and abiotic stress, as well as plant growth and development [31]. Overall, an in-depth study of the mechanisms of *F. graminearum*–plant interaction is necessary to develop new strategies to control FHB. Because there are no reports on the interaction between the effector protein M36 metalloproteinase in *F. graminearum* and wheat CAMTA protein, we conducted a related study.

In this study, we investigated the function of M36 metalloprotease FgFly1 in *F. graminearum*. We found that FgFly1 interacted with wheat TaCAMTA to reduce the disease resistance of the host wheat to achieve the purpose of infection. In addition to its virulence function, FgFly1 also affected the spore germination of *F. graminearum* asexual reproduction and the susceptibility of metal ion stress. We believe that FgFly1 plays an important role in the growth, development and infection of *F. graminearum*.

## 2. Materials and Methods

### 2.1. Culture Conditions for Plants and Fungi

*Nicotiana benthamiana* plants were grown in a glasshouse (24 °C, 70% relative humidity, 16 h light photoperiod). *F. graminearum* strain PH-1 (NRRL 31084), as wild type, was used to construct gene-deletion mutants. Wild-type strains, gene-deletion mutants and complementary strains were cultured on potato dextrose agar medium (PDA), trace-element minimal medium (MM) and complete medium (CM) at 25 °C, and mycelial growth was observed. In a spore reproduction experiment, 5 young fungus dishes from the periphery of 3-day-old colonies were inoculated into 50 mL conical flasks containing 30 mL CMC medium. After 4 days of culture in a shaker (180 rpm) at 25 °C, the number of conidia in each flask was determined under a microscope with a hemocytometer [37]. Each experiment was repeated 3 times. All strains were stored as mycelium in 15% glycerol at −80 °C.

### 2.2. Construction of Gene-Deletion and Complementary Mutants

Construction of gene-deletion and complementary vectors and subsequent transformation of *F. graminearum* was performed by double-joint PCR techniques [38] and polyethylene glycol (PEG)-mediated protoplast transformation [39]. Gene-deletion mutants were identified by PCR detection using relevant primers and further analyzed by Southern blot. We used a designable primer to amplify fragments (Table S1).

### 2.3. Construction of Green Fluorescent Protein (GFP) Vector

In order to construct a FgFly1-GFP vector, the PCR product and the pYF11-GFP-GEN vector digested by XhoI were cotransferred into yeast XK1-25, and the yeast plasmid pYF11-FgFly1-GFP-GEN was obtained. The yeast plasmid pYF11-FgFly1-GFP-GEN was extracted and transformed into *E. coli* DH5α for large-scale amplification of the recombinant plasmid [39]. The FgFly1-GFP fusion vector was transformed to observe the localization of FgFly1. The transformed complementary strains were screened and inoculated on PDA medium with 100 mg/L G418 sulfate. The corresponding primers of the complementary strains were analyzed by PCR and Southern blot. Finally, the green fluorescent protein (GFP) of the obtained strain was observed with a confocal microscope.

### 2.4. Sexual Reproduction and Vegetative Growth Assays

The activated strains were inoculated on carrot agar medium, and 500 μL of 2.5% Tween-20 solution was added to the aerial hyphae of each inoculated strain. The formation of an ascus membrane and the number of perithecia were measured after 15 d of culture under 25 °C black light lamps [40]. In order to determine the susceptibility of strains to stress, the strains were inoculated on PDA medium with different agents and cultured at 25 °C for 3 d; the colony diameter was measured, and the inhibition rate was calculated. The strain mycelial growth inhibition rate (MGIR) was calculated according to the formula MGIR = [(N − C)/C] × 100, where C is the diameter of the control colony, and N is the diameter of the treated colony. Each experiment was independently repeated three times.

### 2.5. Pathogenicity and DON Production Assays

The pathogenicity of corn filaments was analyzed, and the activated strains were inoculated in the middle of young corn filaments and cultured at 25 °C for 4 d with moderate humidity. For pathogenicity analysis of wheat spikelets, conidia of wild-type PH-1, knockout mutants and complementary strains cultured in CMC medium for 4 d were filtered and centrifuged, and the conidia concentration was adjusted to 1 × 10^5^/mL with sterile water. During the flowering stage of wheat, an equal amount of 10 μL of the conidia suspension was injected into the florets of the central part of the susceptible cultivar Xiaoyan 22 with a pipette, and 20 replicates were designed for each strain [41,42]. The wheat was placed neatly on wet filter paper and cultured for 2d at 25–30 °C until the wheat grew 2–3 cm buds. The concentration of spores was adjusted to 1 × 10^5^/mL, and the top 3–5 mm of the wheat buds was cut off with a sharp blade. The absorbent cotton was torn into pieces the size of soybeans, soaked in spore suspension for 5 s and wrapped on the wound on top of the wheat. After moisturizing the culture for 48 h, the absorbable cotton was removed, and the outer epidermis of the wheat coleoptile was removed for microscope observation or continued culture for 5 d to observe the disease situation [41,43]. An emetic toxin ELISA kit (Jiangsu Enzyme Immunity Industry Co., Ltd., YanCheng, China) was used to measure DON production.

### 2.6. qRT—PCR Assays

*Tri* genes were induced with TBI medium and treated in the dark at 25 °C and 180 rpm for 48 h. The mycelia were collected and ground in liquid nitrogen. Total RNA was extracted using RNAiso reagent (TaKaRa Co., Dalian, China), and 1 mg of each RNA sample was subjected to reverse transcription using a HiScriptII QRT Super Mix qPCR kit (Vazyme Biotech, Nanjing, China). The expression level of each gene was determined by quantitative real-time PCR with the primers listed in Table S1. Each experiment was repeated three times independently.

### 2.7. Yeast Two-Hybrid Assays

The gene DNA fragment was inserted into the pBT3-SUC plasmid as a bait vector. The bait and prey vector pPR3-N-cDNA library (Oebiotech, Shanghai, China) was co-transferred into yeast strain NMY51 according to the yeast protocol manual (Clontech, Shanghai, China), and positive clones on SD/-LEU-TRP-HIS-ADE/3-AT medium were selected. In order to confirm the interaction between gene and target, the recombinant pBT3-SUC gene and pPR3-N target were cotransferred into yeast strain NMY51 and grown in SD/-Leu-Trp-His-Ade/3-AT.

### 2.8. BiFC

The gene and target were cloned into BiFC vectors C-YFP and N-YFP, respectively. The constructed bimolecular fluorescent complementary vector was transformed into *Agrobacterium tumefaciens* GV3101 (pSoup-p19) competence, and the correct single clone detected by PCR was cultured in LB medium containing kanapenicillin and rifampicin resistance. The *A. tumefaciens* solution was kept at 28 °C, shaken at 200 rpm and centrifuged at 4000 rpm for 10 min to collect the bacteria, and the supernatant was discarded, and 10 mM MgCl_2_ was added to treat the *Agrobacterium* solution. AS buffer was used to mix c-YFP bacterial solution with N-YFP bacterial solution and P19 bacterial solution according to the OD_600_ ratio of 0.5:0.5:0.3 and then placed in the dark for 2 h before injecting *N. benthamiana*. Fluorescence microscopy was performed after 48–72 h [44].

### 2.9. LUC

The gene and target gene were constructed into n-LUC and c-LUC vectors, respectively, by one-step cloning method. The plasmid was transformed to GV3101 (pSoup-P19), and the monoclonal clones detected by PCR were cultured in LB medium containing kanapenicillin and rifampicin resistance. The *A. tumefaciens* solution was kept at 28 °C, shaken at 200 rpm until the OD_600_ was 1.0–1.3 and centrifuged at 4000 rpm for 10 min to collect the bacteria. Then, the supernatant was discarded, 10 mM MgCl_2_ was added and shaken slightly, followed by the addition of an appropriate amount of infection solution and mixing by pipetting. Then, the solution was kept in the dark for 2 h. The *A. tumefaciens* infection solution was mixed evenly, with a final concentration of 0.2. *N. benthamiana* leaves were injected, marked, cultured in the dark for 12 h and subsequently placed under normal light conditions for 24 h. Then, the *N. benthamiana* leaves injected with Agrobacterium were cut and spread out on the prepared 4% agar plates, and firefly luciflucase substrate was coated against light, and imaging was performed after standing in darkness for 5 min. The exposure time on the plant living molecular labeling imaging instrument was 10 min to collect fluorescence signals [45].

### 2.10. Statistical Analyses

Data are presented as the mean of triplicates. Significance was determined by Fisher exact test at *p* = 0.05.

## 3. Results

### 3.1. Phylogenetic Analysis and Sequence Alignment of Different Fungal Fly1 Proteins and Adcquisition of FgFly1 Deletion Mutants

**Figure 1:**
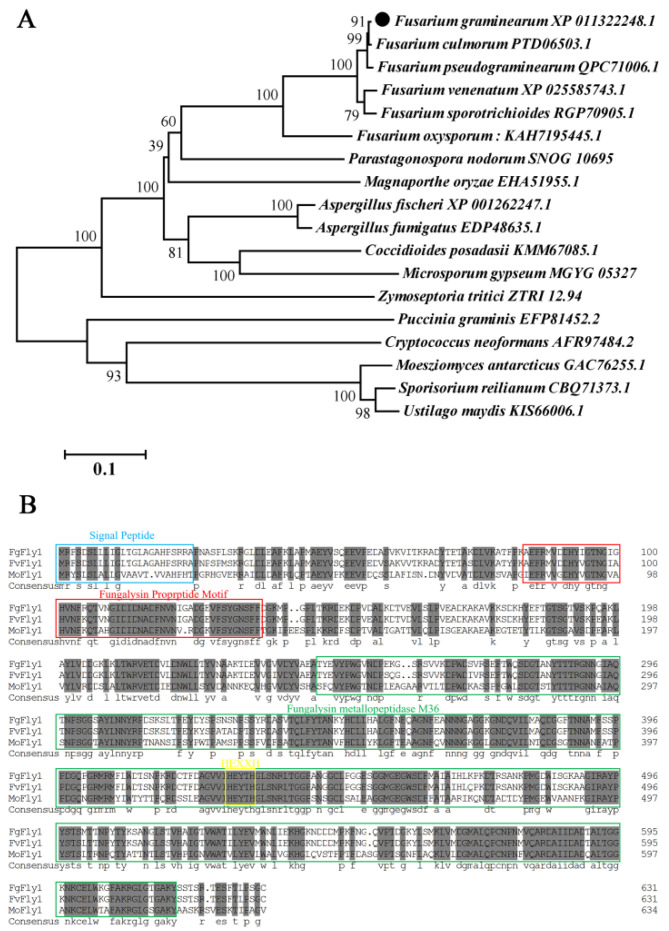
The phylogenetic tree and domain sequence analysis of different fungi Fly1 constructed by Mega5. (**A**) The minimal phylogenetic tree constructed by the full-length sequence alignment of Fly1 homologous proteins in different fungi. The tree was constructed using Mega5 software with 1000 bootstrap times using the minimal evolutionary algorithm. The scale bar indicates the number of replacements at each location. The numbers next to the species names correspond to NCBI protein accession numbers. (**B**) This sequencing of *F. graminearum, Fusarium venenatum* and *Magnaporthe oryzae* was constructed using DNAMAN software. The blue box indicates the signal peptide predicted using SignalP5.0, the red box indicates the FTP domain predicted online using HMMER (https://www.ebi.ac.uk/Tools/hmmer/ accessed on 2 December 2021) and the green box indicates the peptidase M36 domain. The yellow box is the HEXXH site specific to the M36 metalloprotease.

The protein encoded by the FgFly1 gene (FGSG_03467) of *F. graminearum* contains 631 amino acids. Phylogenetic analysis was performed using MEGA software, and sequence alignment was performed using DNAMAN. Species names and accession numbers are as follows: *F. graminearum,* XP_011322248.1; *Fusarium culmorum,* PTD06503.1; *Fusarium pseudograminearum*, QPC71006.1; *Fusarium venenatum*, XP_025585743.1; *Fusarium sporotrichioides*, RGP70905.1; *Aspergillus fischeri*, XP_001262247.1; *Aspergillus fumigatus*, EDP48635.1; *Coccidioides posadasii*, KMM67085.1; *Magnaporthe oryzae*, EHA51955.1; *Zymoseptoria tritici*, ZTRI_12.94; *Puccinia graminis*, EFP81452.2; *Moesziomyces antarcticus*, GAC76255.1; *Sporisorium reilianum*, CBQ71373.1; *Cryptococcus neoformans*, AFR97484.2; *Microsporum gypseum*, MGYG_05327; *Parastagonospora nodorum*, SNOG_10695; *Ustilago maydis*, KIS66006.1. Phylogeny showed that the Fly1 protein of *F. graminearum* is evolutionarily conserved (Figure 1A). In addition, signal peptide prediction (http://www.cbs.dtu.dk/services/SignalP/ accessed on 2 December 2021) Fly1 protein has a 23aa N-terminal signal peptide. Homology alignment (http://pfam.xfam.org/ accessed on 2 December 2021) showed that the FTP domain and peptidase M36 domain, as well as a characteristic HEXXH active site for metalloproteinases, were similar to that of other fungi (Figure 1B).

We constructed a FgFly1 knockout vector by double-joint PCR technique and knocked-out by protoplast transformation. Afterwards, *ΔFgFly1* was identified by PCR and Southern blot (Figure S1).

### 3.2. Deletion of FgFly1 Impairs Asexual Reproduction and Spore Germination of F. graminearum

**Figure 2:**
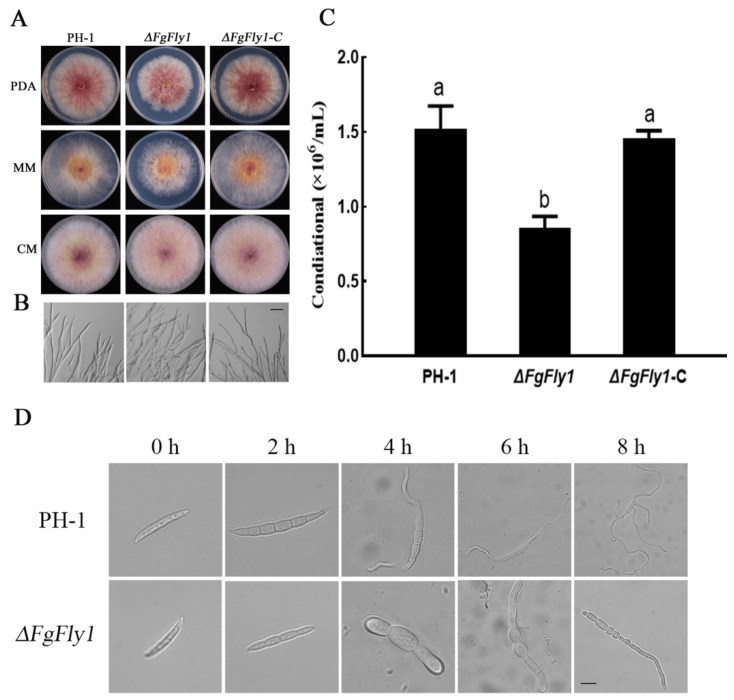
Effects of deletion of FgFly1 on mycelial growth and spores of *F. graminearum.* (**A**)WT PH-1, *∆FgFly1* deletion mutant and *∆FgFly1*-C-complemented strains were grown on PDA, complete medium (CM) and minimal medium (MM), respectively, at 25 °C for 3 d. (**B**) Hyphal growth at the edges of PH-1, *∆FgFly1* and *∆FgFly1*-C colonies. Bar = 20 μm. (**C**) Conidia production by *F. graminearum* strains PH-1, *∆FgFly1* and *∆FgFly1*-C. Conidiation was measured by counting the number of conidia produced in 5-day-old CMC cultures. Bars denote standard deviations from three repeated experiments. The same letter on the bars for each treatment represents no significant difference at *p* = 0.05. (**D**) The morphology of conidia spores was observed under a microscope for 0, 2, 4, 6 and 8 h. Bar = 50 μm.

To investigate the role of FgFly1 in asexual growth, we inoculated WT PH-1, mutant *∆FgFly1* and *∆FgFly1*-C on PDA, CM and MM agar media for culture, respectively. The growth after 3 d is shown in Figure 2A, and the specific data are shown in Table S2. the growth rate of *∆FgFly1* was slightly lower than that of WT PH-1; the hyphae of mutant *∆FgFly1* were more densely branched according to observation with a microscope of the hyphae grown on cellophane (Figure 2B). In addition, the conidial production of each strain was measured after 5 d by inducing asexual conidia on CMC medium, as shown in Figure 2C. The molecular conidial production of mutant *∆FgFly1* was significantly lower than that of wild-type PH-1, and this phenomenon was recovered in the complementary strain *∆FgFly1*-C. The spores were enriched and added to YEPD for germination, and 100 spores were randomly selected at 0, 2, 4, 6 and 8 h to calculate the germination rate. The spore germination rate of WT PH-1 was about 15% at 2 h, and the germination rate of mutant *∆FgFly1* was 3%; the spore germination rate of WT PH-1 was about 72%, and the germination rate of mutant *∆FgFly1* was 31% at 4 h. At 6 h, the spore germination rate of WT ph-1 was about 89%, and that of mutant *∆FgFly1* was 55%. The germination rate of WT PH-1 mutant *∆FgFly1* was 77% at 8 h.

The results in Figure 2D show that the spore germination of the mutant *∆FgFly1* after 4 h, 6 h and 8 h was significantly lower and even slightly deformed compared with that of WT PH-1. These results suggest that FgFly1 is essential for the production of conidia and the process of spore germination in *F. graminearum*.

When assayed for sexual reproduction on carrot agar plates, the *ΔFgFly1* produced perithecia that same as those of wild-type strain PH-1 and *ΔFgFly1*-C (Figure S2). 

### 3.3. ∆FgFly1 Mutants Have Enhanced Tolerance to Metal Cation Stress and Cell-Wall-Related Stress

**Figure 3:**
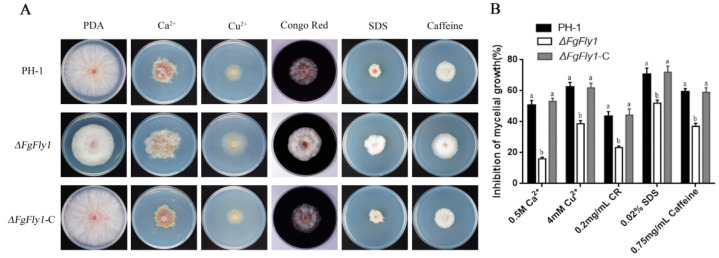
Sensitivity of FgFly1 to metal cations and cell-wall-damaging agents. (**A**) Growth phenotypes of WT PH-1, *∆FgFly1* mutant and *∆FgFly1*-C complement in PDA medium with CaCl_2_, CuSO_4_·5H_2_O, Congo Red, SDS and caffeine, respectively, and cultured at 25 °C for 3 d. (**B**) Statistical analysis of the growth inhibition rate of the strains to the above-mentioned stress. The same letter on the bars for each treatment represents no significant difference at *p* = 0.05.

To verify the effect of FgFly1 on *F. graminearum* tolerance, we examined the susceptibility of *∆FgFly1* mutants to metal cations and cell-wall-damaging agents. WT PH-1, mutant *∆FgFly1* and complementary strain *∆FgFly1*-C were grown on PDA medium containing 0.5 mol/L CaCl_2_, 4 mmol/L CuSO_4_·5H_2_O, 0.2 g/L Congo Red, 0.02% SDS and 0.75 g/L caffeine for 3 d. As shown in (Figure 3A,B), compared with WT PH-1 and *∆FgFly1*-C, *∆FgFly1* exhibited enhanced tolerance to metal cation stress and cell wall disruptors.

### 3.4. Deletion of FgFly1 Leads to Reduced Pathogenicity of F. graminearum

**Figure 4:**
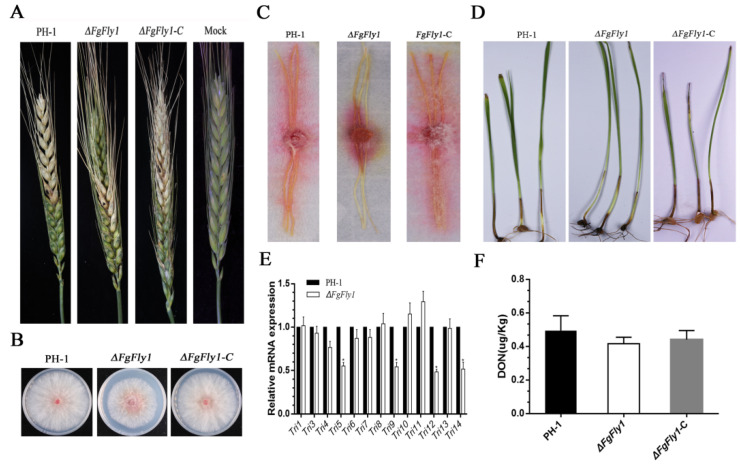
Effect of deletion of FgFly1 on pathogenicity and DON toxin synthesis of *F. graminearum*. (**A**) Disease 15 d after inoculation of flowering wheat ears with conidial suspensions of WT PH-1, deletion mutant *∆FgFly1* and complement strain *∆FgFly1*-C. (**B**) The growth of the pathogenic bacteria isolated from the diseased ear after inoculation on PDA for 3 d. (**C**) The incidence of corn filaments infected with the WT PH-1, deletion mutant *∆FgFly1* and the complement strain *∆FgFly1*-C and inoculated in the middle of maize silk for 7 d. (**D**) Wheat coleoptiles were inoculated with an equal number of fresh conidia of WT PH-1, *∆FgFly1* and *∆FgFly1*-C; brown lesions on the coleoptiles were measured 7 d post inoculation (dpi). Coleoptiles inoculated with distilled water were taken as the control. (**E**) Relative transcription levels of 13 *TRI* genes in WT PH-1 and *∆FgFly1*. For each gene, the expression level in PH-1 was arbitrarily set as 1. Bars denote standard deviations from three repeated experiments. Significant differences compared with PH-1: *, *p* < 0.05. Error bars represent SD of three biological replicates. (**F**) Levels of DON produced by each strain in infected spikelets collected from inoculated wheat heads. Bars denote standard deviations from three repeated experiments.

To investigate the biological function of FgFly1 during wheat infection, we first performed a spore inoculation assay on wheat ears in the flowering stage in the field. On the 15th day of inoculation, the *∆FgFly1* mutant only exhibited symptoms of infection in small patches around the inoculation site, but the infection symptoms did not spread to large areas. The wild type and complementary strain *∆FgFly1*-C showed severe spikelets of infection, which spread to other spikelets of the same plant (Figure 4A). As shown in Figure 4B, the diseased spikelets in the field were isolated and purified, and the growth of *F. graminearum* was the same as that of the wild type, mutant *∆FgFly1* and *∆FgFly1*-C. We also carried out infection experiments on maize filaments. When cultured at 25 °C for 7 d, the WT PH-1 and the complementary strain *∆FgFly1*-C had already infected the whole maize filaments, but the mutant *∆FgFly1* did not spread significantly (Figure 4C). In addition, we also performed coleoptile infection experiments in which the ectodermal stems inoculated with PH-1 and *∆FgFly1*-C turned significantly dark brown after 7 d post-inoculation. In contrast, the lesion size of the deletion mutant *∆FgFly1* was relatively small. (Figure 4D). The specific data from Figure 4A,C,D are presented in Table S2. Compared with PH-1, the hyphal growth rate of *∆FgFly1* decreased by only 20%, whereas FgFly1 was active during infection, indicating that the reduced growth rate was not the key factor associated with the reduced virulence of the modified mutant.

Deoxynivalenol (DON) produced by *Fusarium* is considered a key virulence factor of *F. graminearum* [46]. Plant infection experiments showed that deletion of FgFly1 reduced the virulence of *F. graminearum*; therefore, we measured DON produced by the three strains with a DON toxin ELISA kit. Compared with wild type, the ability of mutant *∆FgFly1* to produce DON toxin did not change significantly (Figure 4F). To further confirm this result, we determined the expression levels of key biosynthetic genes of trichocelene by qRT-PCR, and the results are shown in Figure 4E. Compared with wild type, the expression levels of the *Tri* gene family in *∆FgFly1* were not significantly changed. In conclusion, FgFly1 may affect the pathogenicity-related pathways of wheat disease resistance. 

### 3.5. The Signal Peptide of FgFly1 Has a Secretory Function, and Overexpression Causes Allergic Necrosis in N. benthamiana

**Figure 5:**
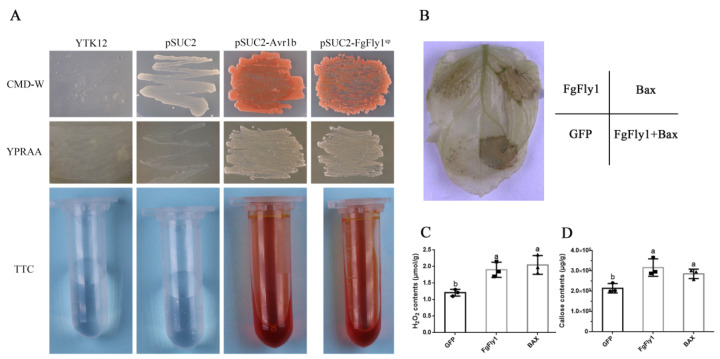
Effects of FgFly1 on the accumulation of H_2_O_2_ and callose in *N. benthamiana* and analysis of its signal peptide secretion function. (**A**) The yeast YTK12 strain carrying the FgFly1 signal peptide fragment fused in the pSUC2 vector were able to grow in both the CMD-W and YPRAA media and induced the TTC red reaction. (**B**) FgFly1, like Bax, can induce cell death in *N. benthamiana*, which was decolorized with alcohol and photographed 5 d after infection. (**C**) The H_2_O_2_ content was measured in *N. benthamiana* leaves collected for 2 d. Bars denote standard deviations from three repeated experiments. The same letter on the bars for each treatment represents no significant difference at *p* = 0.05. (**D**) 2 d *N. benthamiana* leaves were collected to determine H_2_O_2_ content. Bars denote standard deviations from three repeated experiments. The same letter on the bars for each treatment represents no significant difference at *p* = 0.05.

In order to verify whether the FgFly1 signal peptide has a secretory function, we amplified the full-length signal peptide (amino acids 1-23), constructed pSUC2-FgFly1^sp^ and used the sucrose invertase secretion system of the YTK12 strain to verify the N-terminal signal peptide of FgFly1. It has a secretory function [47]. As shown in Figure 5A, the YTK12 strain was unable to synthesize tryptophan with sucrose invertase, so it is not able to grow on CMD-W medium lacking tryptophan and YPRAA, which contains only raffinose as a carbon source. Because the recombinant plasmid replaced the signal peptide of the yeast sucrose invertase with the target signal peptide FgFly1^SP^ and only the secreted sucrose invertase was able to use the raffinose on YPRAA, the transformed strain was able to grow on YPRAA and induced the TTC red reaction, indicating that the N-terminal signal peptide of FgFly1 has a secretory function. In order to study the function of FgFly1, we ligated its full-length coding sequence into a pBin vector and transiently expressed the FgFly1 gene in *N. benthamiana* leaves using *A. tumefaciens* infiltration technology. We found that transient expression of FgFly1 promoted *N. benthamiana* cell death, as well as the apoptosis-promoting gene Bax (Figure 5B). H_2_O_2_ and callose were measured in 48 h *N. benthamiana* leaves. FgFly1 and Bax produced significantly higher H_2_O_2_ and callus compared to the GFP control vector(Figure 5C,D).

### 3.6. Interaction between FgFly1 and TaCAMTA

**Figure 6:**
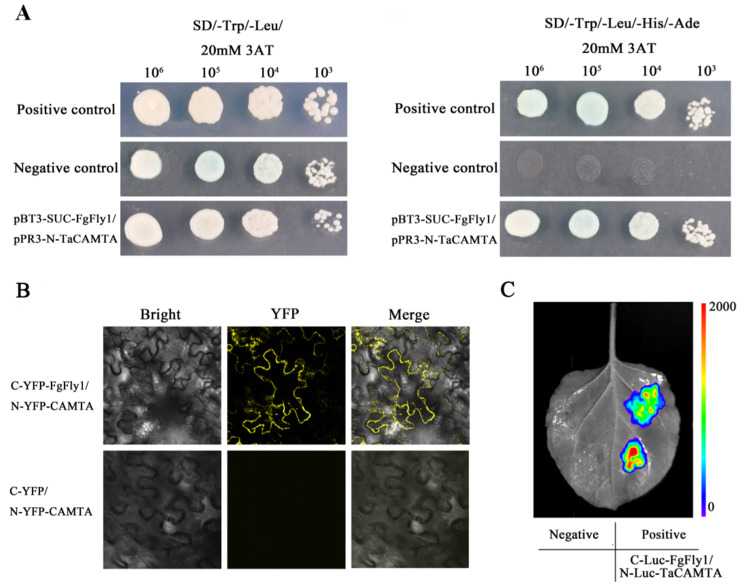
Verification of the interaction between FgFly1 and TaCAMTA. (**A**) Using the Yeast two-hybrid system, pNubG-Fe65/pTSU2-APP, pBT3-SUC-FgFly1/pPR3-N-TaCAMTA and pBT3-SUC-FgFly1/pPR3-N were cotransformed into yeast NMY51. All combinations can be grown on SD/-Trp/-Leu double-deficient medium, but only positive controls pNubG-Fe65/pTSU2-APP and pBT3-SUC-FgFly1/pPR3-N-TaCAMTA can grow on SD/-Trp/-Leu/-His/-Ade four-deficiency medium. (**B**) Agrobacterium containing C-YFP-FgFly1/N-YFP-TaCAMATA and C-YFP/N-YFP-TaCAMTA was mixed into *N. benthamiana* leaves, and the fluorescence signal in the cells was detected under a fluorescence microscope 48 h after injection. (**C**) Agrobacterium containing C-Luc-FgFly1/N-Luc-TaCAMTA was mixed and injected into *N. benthamiana* leaves, and the fluorescence signal was detected by a plant in vivo molecular imaging system 48 h after injection.

We obtained the candidate target protein TaCAMTA of FgFly1 by screening the wheat yeast library. To further verify the interaction between FgFly1 and TaCAMTA, we cotransformed the plasmid pPR3-N-CAMTA/pBT3-SUC-Fly1 into an NMY51 yeast-competent cell for Yeast two-hybrid verification. (pNubG-Fe65/pTSU2-APP) was used as positive control, and pBT3-SUC-Fly1/pPR3-N was used as negative control. Single colonies growing on SD/-Trp/-Leu-deficient medium were picked and diluted at 10^6^, 10^5^ and 10^4^ gradients, respectively, and then spotted on SD/-Trp/-Leu/-His/-Ade/AbA plates. The tested yeasts were found to grow normally (Figure 6A), indicating that the candidate target protein TaCAMTA and the effector protein FgFly1 could interact through the Yeast two-hybrid system. *A. tumefaciens* containing C-YFP-Fly1 and N-YFP-TaCAMTA vectors were constructed, mixed in equal proportions and coinjected into *N. benthamiana* leaves by *A. tumefaciens* infiltration technology. The 2 d infected leaves were observed by laser confocal microscopy for YFP fluorescence signal, and C-YFP/N-YFP-TaCAMTA was used as a negative control. The results are shown in Figure 6B; no fluorescence signal was detected in the negative control under laser confocal microscopy, and a YFP fluorescence signal was detected in C-YFP-Fly1/N-YFP-TaCAMTA, indicating that there was an interaction between FgFly1 and TaCAMTA. *A. tumefaciens* containing C-Luc-FgFly1 and N-Luc-TaCAMTA were constructed, mixed in equal proportions and coinjected into *N. benthamiana* leaves by *A. tumefaciens* infiltration technology, and the infected leaves were taken for 36 h using a plant living molecular imaging system. The results are shown in Figure 6C. C-Luc-FgFly1/N-Luc-TaCAMTA has the same fluorescence as the positive control, but no fluorescence appeared in the negative control, indicating that there was an interaction between FgFly1 and TaCAMTA.

### 3.7. CAMTA Functional Verification

**Figure 7:**
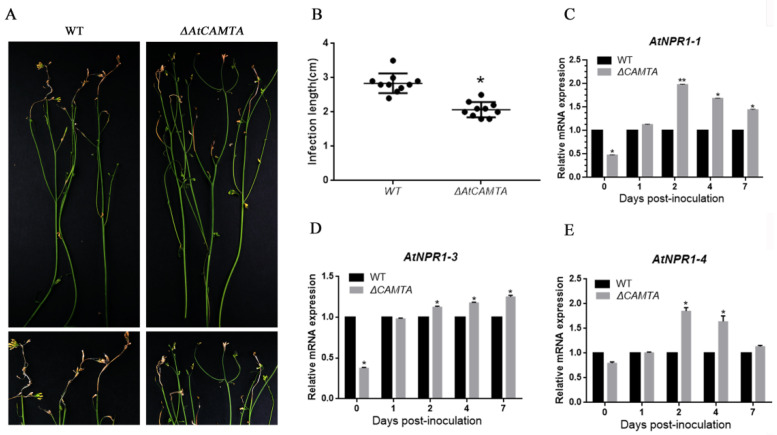
Functional validation of CAMTA in *A. thaliana*. (**A**) The stamens of *A. thaliana* photographed 7 d after *F. graminearum* infection. (**B**) *A. thaliana* lesion length; Significant differences compared with WT: *, *p* < 0.05. Error bars represent SD of three biological replicates. (**C**) Expression of the *NPR1-1* gene when *F. graminearum* infects *A. thaliana*. Significant differences compared with WT: *, *p* < 0.05; **, *p* < 0.01. Error bars represent SD of three biological replicates. (**D**) Expression of the *NPR1-3* gene when *F. graminearum* infects *A. thaliana*. Significant differences compared with WT: *, *p* < 0.05. Error bars represent SD of three biological replicates. (**E**) Expression of the *NPR1-4* gene when *F. graminearum* infects *A. thaliana*. Significant differences compared with WT: *, *p* < 0.05. Error bars represent SD of three biological replicates.

CAMTA plays an important regulatory role in plant biotic stress, abiotic stress, and plant growth and development as a CaM-binding protein in response to a series of environmental stresses, such as drought, salt and cold and hormonal signals, such as abscisic acid, ethylene and growth hormone [48]. Therefore, we used the *A. thaliana ∆CAMTA* mutant to verify the function of CAMTA in disease resistance. *A. thaliana* was infected with wild-type PH-1 spore fluid in the flowering stage, and the results are shown in Figure 7A. The disease resistance of CAMTA-deficient *A. thaliana* was enhanced relative to wild-type *A. thaliana*. The disease spot lengths of 10 *A. thaliana* plants were counted; the CAMTA-deficient mutant had significantly shorter spot lengths than the wild type, as shown in Figure 7B. CAMTA has been reported to negatively regulate NPR1-mediated immune response [49], so we collected *A. thaliana* at different infection stages of *F. graminearum* to determine the expression levels of NPR1-1, NPR1-3 and NPR1-4. Compared to WT, AtNPR1-1, AtNPR1-3 and AtNPR1-4 were found to be significantly up regulated in the *ΔAtCAMTA* mutant after 24 h. In conclusion, CAMTA is a susceptibility gene that plays a negative regulatory role in mediating the expression of the disease-resistance gene NPR1.

## 4. Discussion

In the process of plant–pathogen coevolution, a complex interaction relationship is formed. Plants establish many recognition and resistance mechanisms to organize and limit the infection of pathogens. Pathogens also form a variety of pathogenic mechanisms in order to avoid or overcome plant disease-resistance mechanisms, such as the formation of special infection structures, the secretion of a variety of hydrolytic enzymes, the production of host-selective toxins and the detoxification of plant-resistant substances [50,51,52].

Among fungal pathogens, metalloproteases (MEPs) are related to the autotoxicity of fungal pathogens [53]. Secreted metalloproteases are important pathogenic factors for many fungal diseases and cause damage to hosts to varying degrees. Sanz-Martin et al. (2016) found that the fungal hemolysin metalloproteinase gene *Cgfl* of *Colletotrichum graminicola* was also involved in fungal virulence to maize [28]. *Avr-Pita*, a metalloproteinase in *Magnaporthe grisea*, has been proven to be a specific pathogenic factor that can directly bind to plant resistance gene products, trigger signal cascade and thus produce resistance [29]. Although *F. graminearum* has been thoroughly studied, it is still unclear whether the M36 metalloprotease of *F. graminearum* plays key a role in host resistance. This study revealed the function of M36 metalloprotease in the development and infection of *F. graminearum*.

Based on homology BLAST search, a single-copy gene encoding M36 metallopeptidase was found in *F. graminearum*. FgFly1, similarly to other fungi, contains signal peptide, fungalysin propeptide motif, fungalysin metallopeptidase (M36) and HEXXH structure. The reported M36 metalloprotease of maize powdery mildew affects the differentiation and virulence of conidia of maize powdery mildew and has the ability to cleave the maize chitinase [54]. In Fusarium verticillioides, FvFly1 was also found to have the ability to truncate chitinase [55]. In our study, the deletion of *F. graminearum* FgFly1 affected asexual reproduction, as well as virulence (Figure 2 and Figure 4). The sensitivity of *F. graminearum* to cell-wall-related stress decreased after the deletion of FgFly1 (Figure 3). Therefore, we suspected that FgFly1 also interacted with wheat type-IV chitinase and carried out point-to-point verification, although the verification was not successful. We did not rule out the possibility of FgFly1 interacting with other types of chitinases, so we screened the wheat cDNA library, but no chitinase-related targets were found in the results. Interestingly, we found TaCAMTA in selected targets and validated the interaction (Figure 6). CAMTA is a kind of important calmodulin-binding transcription factor. As a CaM-binding protein in multicellular eukaryotes, CAMTA responds to a series of environmental stress and hormone signals and plays an important regulatory role in plant stress, abiotic stress, and plant growth and development. Studies have shown that the CAMTA gene family is associated with plant innate immune signals [36]. In rice, oScbT (CAMTA member) plays a role in resistance to *Xanthomonas oryzae* pv. *oryzae* and *Magnaporthe grisea* [56]. Peiguo Yuan et al. (2020) found that CAMTA directly regulated the transcription of the NPR1 gene and played a key role in SA-mediated immune response [49]. Similarly to our results, the NPR1 gene was significantly upregulated in CAMTA-deficient Arabidopsis mutants, and the disease resistance of *ΔAtCAMTA* was significantly enhanced compared with wild type (Figure 7). Consistent with previous results, *CAMTA* is a susceptible gene that can negatively regulate the expression of *NPR1*.

In summary, we found that FgFly1 was involved in the asexual reproduction of *F. graminearum*, reducing plant disease resistance and enhancing its infection ability through interaction with CAMTA. This is the first instance of identification of the target CAMTA of *F. graminearum* M36 metalloprotease. In contrast to the functions of M36 metalloprotease in other fungi, these results may help to elucidate other filamentous fungal infection hosts.

## 5. Conclusions

The results presented here indicate that *F. graminearum* M36 metalloproteinase (FgFly1) is an effector with certain functions. As an effector within *F. graminearum*, *ΔFgFly1* affected sexual reproduction in *F. graminearum* and influenced the sensitivity to Ca^2+^, Cu^2+^ and cell-wall-related stresses. Interestingly, *ΔFgFly1* had no significant effect on DON toxin production or the expression level of the *Tri* gene family, but pathogenicity was significantly reduced relative to PH-1 and *ΔFgFly1*-C, so we concluded that FgFly1 affected wheat, enhancing its pathogenicity. We then screened TaCAMTA for interactions with FgFly1 by Y2H and verified the interactions using Bi-FC and Luc. Inoculation of *A. thaliana* CAMTA deletion mutant with *F. graminearum* on pistil revealed that the disease resistance of *A. thaliana* was enhanced, and the expression level of *A. thaliana* NPR1 was significantly upregulated, proving that CAMTA is a disease-susceptibility gene. We tentatively concluded that FgFly1 interacts with the susceptibility gene CAMTA to promote its expression to enhance pathogenicity. In the present study, we explored the function of FgFly1 at the transcriptional level to further understand the pathogenesis of the effector FgFly1, which is important for the exploration of the pathogenesis of *F. graminearum*.

## Data Availability

The data presented in this study are available on request from the corresponding author.

## Supplementary Materials

The following supporting information can be downloaded at: https://www.mdpi.com/article/10.3390/jof8070726/s1, Table S1: PCR primers used in this study; Table S2. Growth rate, average disease severity and plant infection between different types of strains. Figure S1: PutativeFly1 mutant was screened by PCR analysis, and putative Fly1 mutants were screened by Southern blot analysis; Figure S2: Analysis of ascospores discharge from PH-1, *ΔFgFly1* and *ΔFgFly1-C* on carrot plate for 15 d.
