# Supplementary material for: Functional Characterization of the M36 Metalloprotease FgFly1 in Fusarium graminearum
Source: J Fungi (Basel). 2022 Jul 12;8(7):726. doi: 10.3390/jof8070726 (PMC9316299; doi:10.3390/jof8070726)
Supplement: Supplementary file 1 [file jof-08-00726-s001.zip › jof-1795972-supplementary.pdf]

Supplementary

Table S1. PCR primers used in this study.

NAME	SEQUENCE(5′-3′)
FLY1-UP-F	TCTTGTGTCATCAGGCACTG
FLY1-UP-R	CAAAATAGGCATTGATGTGTTGACCTCCGTTGAGAACGAGTGAGGATT
FLY1-DOWN-F	CTCGTCCGAGGGCAAAGGAATAGAGTAGATATGATACGATGGAATGTT
FLY1-DOWN-R	AGAGTGGAGCGCTGCTCAGA
FLY1-ID-F	CCATGATCGCGTGCTTGAAC
FLY1-ID-R	CGCTGGTTATCAATCTCTGC
FLY1-NEST-F	GAAGCTCGCCCGGTAGCGTC
FLY1-NEST-R	CCAGTACAACGCTTTCCACC
NP-FLY1-GFP-F	ACTCACTATAGGGCGAATTGGGTACTCAAATTGGTTAGACGAGATATAGGCTCTTG
NP-FLY1-GFP-R	CACCACCCCGGTGAACAGCTCCTCGCCCTTGCTCACGCAGCCAGAAGGCAGGGTGAA
ID-Fly1-GFP-F	TCCATGAAGCTCGTCATGGAC
ID-Fly1-GFP-R	GTCAGCTTGCCGTAGGTGGCA
CYFP-FLY1-F	CTAGTCGACTCTAGCCTCGAGATGGCACCCAACGCATCGCC
CYFP-FLY1-R	ATCGTATGGGTACATCCTAGGGCAGCCAGAAGGCAGGGTGAA
NYFP-CABT-F	TCTGAGGAGGATCTTCCTAGGATGAATGTGATTCAATATTA
NYFP-CABT-R	GGGAGGCCTGGATCGACTAGTGCAGCAAATACATACACCAG
PPR3N-FLY1-F	GACGCGTGGCCATTACGGCCATGCGCTTCTCCGATAGTCT
PPR3N-FLY1-R	CTCGAGAGGCCGAGGCGGCCGGCAGCCAGAAGGCAGGGTGAA
PBT3-FLY1-F	ATGTAATGGCCATTACGGCCATGCGCTTCTCCGATAGTCT
PBT3-FLY1-R	TGCAGATGGCCGAGGCGGCCCCGCAGCCAGAAGGCAGGGTGAA
PBT3-ΔSPFLY1-F	ATGTAATGGCCATTACGGCCATGGCACCCAACGCATCGCC
PPR3-CABT-F	GACGCGTGGCCATTACGGCCATGAATGTGATTCAATATTA
PPR3-CABT-R	CTCGAGAGGCCGAGGCGGCCGGCAGCAAATACATACACCAG
PBT3-CABT-F	ATGTAATGGCCATTACGGCCATGAATGTGATTCAATATTA
PBT3-CABT-R	TGCAGATGGCCGAGGCGGCCCCGCAGCAAATACATACACCAG
NLUC-CABT-F	GAACACGGGGGACGAGCTCGGTACCATGAATGTGATTCAATATTA
NLUC-CABT-R	GCCTCAGTCGACGCGTTGTGGATCCGCAGCAAATACATACACCAG
CLUC-FLY1-F	CTCGTACGCGTCCCGGGGCGGTACCATGGCACCCAACGCATCGCC
CLUC-FLY1-R	GCCTCAGTCGACGCGTTGTGGATCCGCAGCCAGAAGGCAGGGTGAA
PSUC2-FLY1-F	TCCAAGCTCGGAATTTTAATTAAGAATTCATGCGCTTCTCCGATAGTCT
PSUC2-FLY1-R	CGACTCACTATAGGGAGAACCTCGAGATGGGCACCGGCCAGGCCGG
PBIN-NSP-FLY1-F	CCCCGGGGTCGACGGATCCATGGCACCCAACGCATCGCC
PBIN-FLY1-F	CCCCGGGGTCGACGGATCCATGCGCTTCTCCGATAGTCT
PBIN-FLY1-R	CTCTAGTTCATCTAGAGGATCCGCAGCCAGAAGGCAGGGTGA
CLUC-JC-F	GAGTTGTGTTTGTGGACGAAGTACC
CLUC-JC-R	TTCCATTTCACAGTTCGATAGCG
NLUC-JC-F	TTGGAGAGAACACGGGGGACG
NLUC-JC-F	TTGGAGAGAACACGGGGGACG
NLUC-JC-R	CAGGGCGTATCTCTTCATAGCC
RT-TRI1-F	GATGTTCCTTCTCGACAGCGT
RT-TRI1-R	CACTGGTCGAAGATAGCTGG
RT-TRI3-F	TGTTACGATCAATGGCTTGG
RT-TRI3-R	TCCTCGTTGTAGTTTGCATCA

RT-TRI4-F	ACGTGTGGCTACTCAGGAGA
RT-TRI4-R	TGGAATTGCCTTGGGGTA
RT-TRI5-F	ATGGCGGATCTATCTATTCAC
RT-TRI5-R	CCATTCATACGACGAAGGAAT
RT-TRI6-F	AAATGCCCATTCCCTAGTTG
RT-TRI6-R	ATCTCGCATGTTATCCACCCT
RT-TRI7-F	TACCGTCGTCTTCAAAACCA
RT-TRI7-R	ACGCCAATGGTGTTCACAAA
RT-TRI8-F	ATATAACGGTACCCCCAGATG
RT-TRI8-R	TGTTTGTAGGACACTTCCGGT
RT-TRI9-F	CCGCTAAACTGATCGACTCAT
RT-TRI9-R	CCCATATGGTAGCGCATAAA
RT-TRI10-F	TCCCAACCTTTCAGAGGTTCA
RT-TRI10-R	TGATCCGTCAAGTCTTCCCAT
RT-TRI11-F	TGAGAACGACATGTGGGCAAT
RT-TRI11-R	AGGCTTGTTCCATGCAAGAT
RT-TRI12-F	ACGAACAGCACTGCTACGGT
RT-TRI12-R	TTCCTGCTTGTGACTCCAAT
RT-TRI13-F	ACGCAGATCCTGGGATATCA
RT-TRI13-R	CAGCCCAGTATTTTGCCAAA
RT-TRI14-F	AACTCCCGTTGTGATCAAGCA
RT-TRI14-R	AACAGTAATGTTGGCACCGT
RT-ACTIN-F	ATCCACGTCACCACTTTCAA
RT-ACTIN-R	TGCCTTGAGATCCACATTTG

Table S2. Growth rate, average disease severity and plant infection between different types of strain

Strain	Growth rate (mm d^{-1})[1)]	Average disease severity[2)]	Maize silk lesion length (cm)[3)]	Coleoptile Lesion length(cm)[4)]
PH-1	11.5±0.3a	3.17a	10±0.2a	1.2±0.12a
ΔFgFly1	9.2±0.2b	1.13b	4.2±0.1b	0.3±0.05b
ΔFgFly1-C	11.4±0.3a	3.20a	9.8±0.3a	1.2±0.10a

1) Daily extension in colony radius on potato dextrose agar (PDA) plates.

2) Average disease severity was rated by the disease severity of symptomatic spikelets per head at 14 days post-inoculation. (Total 30 spikes)

3) The average lesion length 7 days after infection of maize silk. (Total 10 spikes)

4) The average lesion length 7 days after infection of coleoptile. (Total 10 spikes)

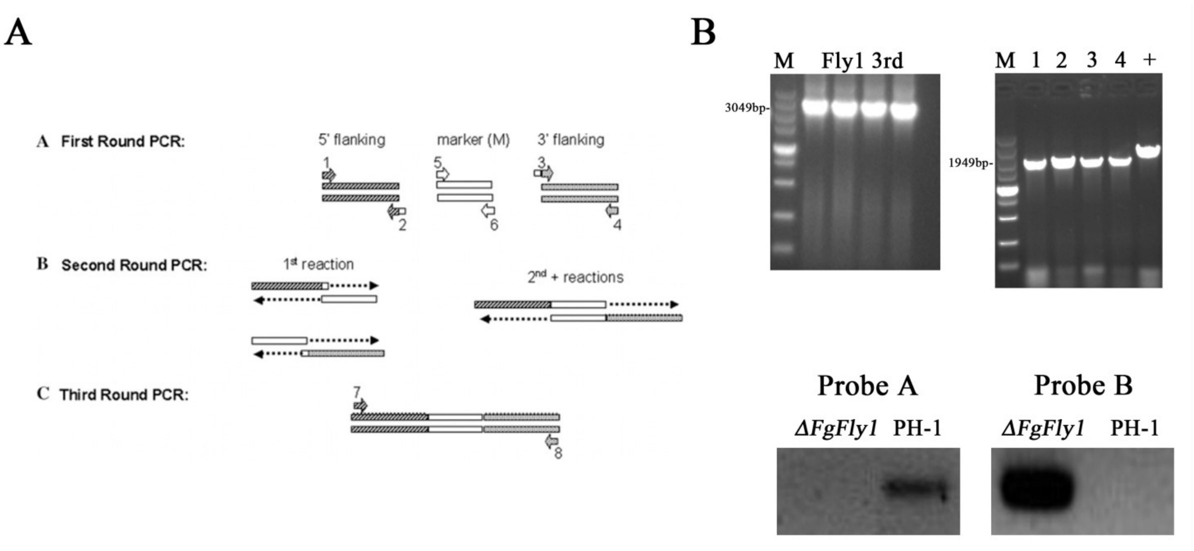

Figure S1.A. Schematic diagram of Double-joint PCR. B. Fly1 mutants were screened by PCR analysis and Southern blot.

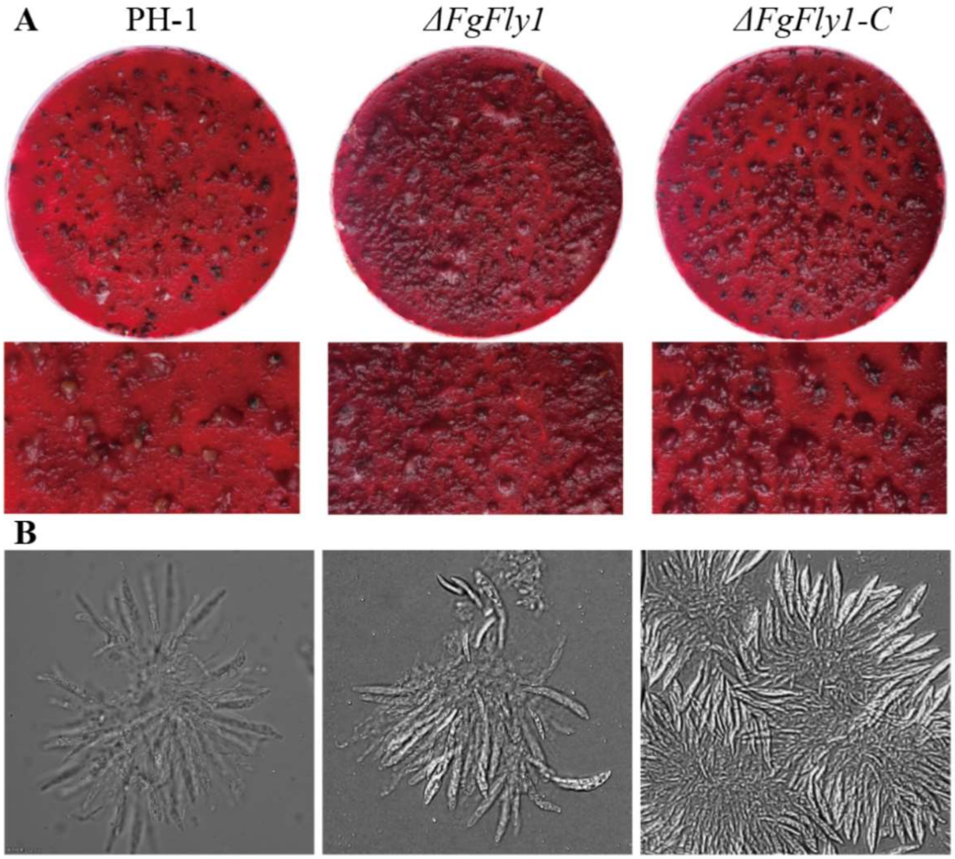

Figure S2. Analysis of ascospores discharge from PH-1, ΔFgFly1 and ΔFgFly1-C on carrot plate for 15 d. A. Carrot agar cultures of the PH-1, ΔFgFly1 and ΔFgFly1-C were examined 15 d post-fertilization (dpf). B. Ascospores of the PH-1, ΔFgFly1 and ΔFgFly1-C were examined by differential interference contrast (DIC). Bar= 10 μm.
